# All-cause and cardiovascular mortality in dual sensory impairment patients: A meta-analysis of cohort studies

**DOI:** 10.7189/jogh.14.04258

**Published:** 2024-11-29

**Authors:** Shuyi Liu, Tao Qin, Don O Kikkawa, Wei Lu

**Affiliations:** 1 Department of Ophthalmology, The Second Hospital of Dalian Medical University, Dalian, China; 2 Office of teaching affairs, Dalian Medical University, Dalian, China; 3 Department of Ophthalmology, Shiley Eye Institute, San Diego, California, USA; 4 Department of International Education College, Dalian Medical University, Dalian, China

## Abstract

**Background:**

This meta-analysis is to determine the risk of all-cause mortality and cardiovascular mortality of dual sensory impairment (DSI).

**Methods:**

Relevant cohort studies were searched in Medline with PubMed, Cochrane Library, and EMBASE databases. The quality of the included studies was assessed based on the Newcastle-Ottawa Quality Assessment Scale (NOS). STATA software (USA) was used to conduct statistical analyses. To determine the source of heterogeneity, subgroup and sensitivity analyses were carried out. Funnel plots and the Egger's test were used for detecting publication bias.

**Results:**

This meta-analysis incorporated 12 cohort studies (1992–2024), containing 310 211 patients. Pooled analysis showed that DSI patients had a higher risk of all-cause mortality (hazard ratio (HR) = 1.442; 95% confidence interval (CI) = 1.303–1.596, *I*^2^ = 49.5%, *P* < 0.001), and cardiovascular mortality (HR = 1.832; 95% CI = 1.343–2.500, *I*^2^ = 0%, *P* < 0.001). Subgroup analyses on sex and territory type revealed that DSI were all associated with an increased risk of all-cause mortality.

**Conclusions:**

This study shows that DSI is linked to higher risks of all-cause and cardiovascular mortality, suggesting that DSI should be regarded as an independent mortality risk factor. Physicians treating individuals with DSI should assess its impact on life expectancy.

**Registration:**

The protocol was previously registered on the International Prospective Register of Systematic Reviews (PROSPERO) platform (CRD42024527256).

Sensory impairment, including vision impairment (VI) and hearing impairment (HI), is age-related condition [1]. As reported by the World Health Organization in 2019, approximately 2.2 and 1.5 billion people worldwide affects by VI and HI, respectively [2,3]. These numbers are expected to increase in the coming years due to a growing and aging population [4]. Concurrent VI and HI is termed as dual sensory impairment (DSI), and related to a range of functional or clinical complications, including frailty, hip fracture, neuronal degeneration, and cognitive impairment [5,6]. Meanwhile, DSI can also result in a partial or entirely disconnection from society, increasing depression risk and decreasing life quality [7].

Although it is widely recognised that sensory function has a significant association with health and quality of life, a growing body of evidence suggests that sensory impairment may also be an understudied indicator of elevated risk for mortality [8]. However, investigations on the relation between sensory impairment and mortality came to the opposite conclusion [9–12]. These contradictory results may be mainly due to residual confounding by failure to mutually adjust for HI and VI. Since HI and VI share risk factors and frequently occur together rather than separately, and DSI patients accumulates more negative physical functioning and mortality outcomes, it is necessary to take them into consideration at the same time. However, most previous studies focused on the relation of sensory impairment and mortality, HI or VI singly, not together. Therefore, a thorough assessment of the overall risk for mortality in DSI is required. Predicting mortality risks has substantial implications for prevention, identification, and treatment in DSI individuals, which avoiding such hazards and lowering DSI-related mortality. Therefore, we conducted a study to determine the all-cause and cardiovascular mortality risks associated with DSI.

## METHODS

The present study adhered to the Preferred Reporting Items for Systematic Reviews and Meta-Analyses (PRISMA) guidelines, and the protocol was previously registered on the International Prospective Register of Systematic Reviews (PROSPERO) platform (CRD42024527256) [13].

### Data sources and searches

We conducted a systematic search of databases including Medline with PubMed, Cochrane Library, and EMBASE until 22 March 2024. The searching utilised both medical subject headings and keywords. The terms included ‘sensory impairment,’ ‘mortality,’ and their related keywords (**Online Supplementary document**). Only English language publications are eligible. We personally examined the references of the eligible studies and other published meta-analysis for additional relevant studies.

### Inclusion criteria

The eligible studies required to match the criteria listed below:

(1) cohort study

(2) patients with a diagnosis of DSI aged 18 or older

(3) healthy individuals or non-DSI patients in the control group

(4) assessments of the relation of DSI with mortality risk

(5) mortality risk as the primary outcome, reported as hazard ratio (HR), or relative risk (RR) and its corresponding 95% confidence interval (CI).

When studies published data on the same population, we chose the study with the longest follow-up or the largest number of individuals.

### Exclusion criteria

The exclusion criteria were listed as follows:

(1) duplicate publication

(2) study protocols, reviews, case reports, conference abstracts, or letters

(3) no results of interest.

### Study selection

Two authors (SY Liu and T Qin) separately assessed the titles and abstracts of all records following predetermined criteria. Duplicate and irrelevant articles were removed after initial assessments. Then we downloaded the article and conducted a thorough examination to confirm all eligible studies. If there was a disagreement, we discussed it with W Lu.

### Data extraction

SY Liu and T Qin separately collected the following information: first author, publication year, nation or region, study type, sample size, study period, age, diagnosis of sensory impairment and mortality, and confounding factors. W Lu examined the data. If there was a disagreement, we discussed it with W Lu.

### Risk of bias assessment

We employed the Newcastle-Ottawa Quality Assessment Scale (NOS) to assess study quality across three dimensions: selection, comparability, and outcome [14]. Scores were categorised as follows: low quality (0–3), moderate quality (4–6), and high quality (7–9).

### Statistical analysis

We analysed the data using STATA software (version 14.0, Illinois, USA, 1985) and collected adjusted HR and 95% CI from the included studies to assess the association between DSI and mortality. Heterogeneity was assessed using the χ^2^ test and *I*^2^ value, with a random-effects model employed due to clinical heterogeneity. Sensitivity analysis evaluated overall effect reliability, while funnel plots and Egger's regression test were used to examine publication bias. Subgroup analyses were conducted based on sex and territory type.

## RESULTS

### Search results

**Figure 1:**
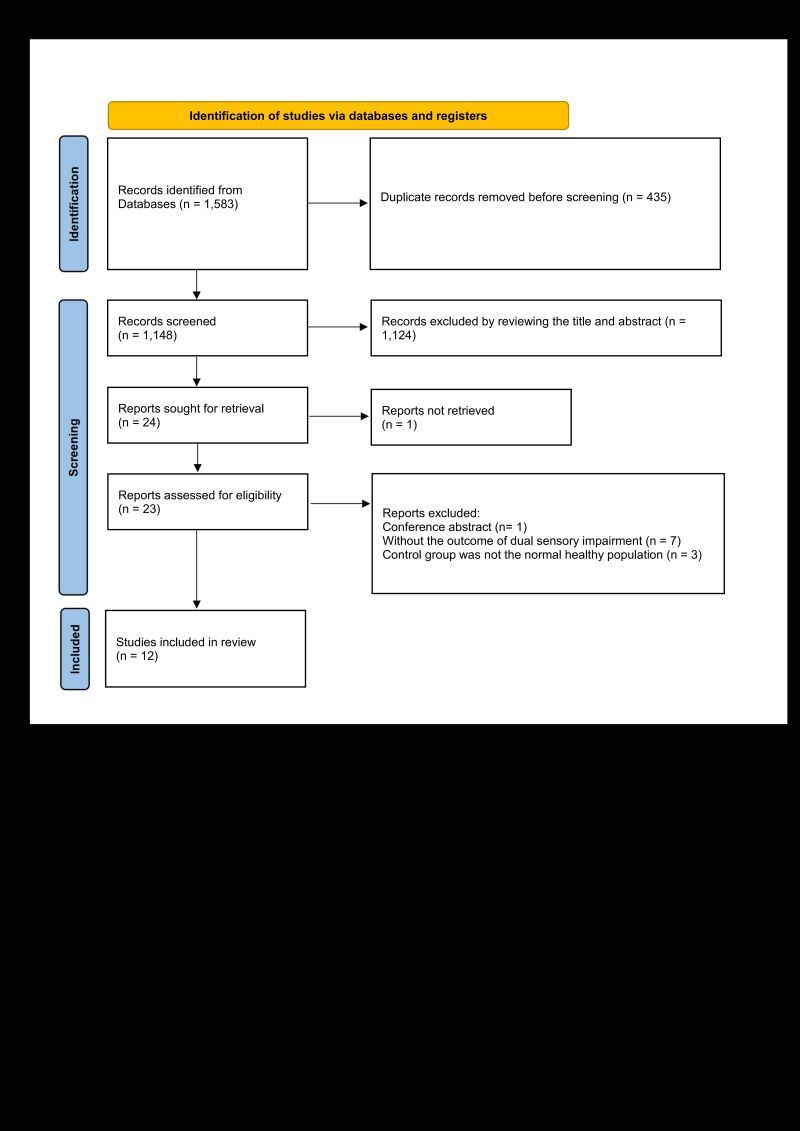
Literature screening flowchart.

We collected 1583 articles, and 435 duplicate studies were discarded. A total of 1124 articles were discarded after examining the title and abstract. Then 11 studies were excluded after screening the full-text [15–25]. Twelve studies were finally included in this systematic review [26–37]. The selection process is showed in **Figure 1**.

### Study characteristics

This meta-analysis included 12 cohort studies involving 310 211 individuals. The published year of these studies were 1992–2024. The sample size of the included studies was ranged from 1405 to 116 796 individuals. The diagnosis of DSI in eight studies was the International Classification of Diseases-9 or 10 (ICD-9 or 10) codes or assessed by the interviewers [27–30,34–37]. The diagnosis of mortality in three studies [28–30] was the ICD-9 or 10 codes. The adjusted confounding factors in the included studies were slightly different. The detailed information of the 12 cohort studies were summarised in **Table 1**.

**Table 1:** Characteristics of cohort studies included in the meta-analysis

Source	Sample Size	MD follow-up, y	Age, y	Male, %	Diagnosis of sensory impairment	Diagnosis of mortality	Confounders adjusted	NOS scores
Laforge RG, 1992, America	1405	1	NA	38.3	Self-reported.	/	Sex and age	4
Reuben DB, 1999, America	5646	10 (x̄)	65.8 ± 5.3 (55–74)	47.5	Self-reported or measured (VI: 20/40 or worse in the better eye; HI: 40-dB loss at either the 1- or 2-kHz frequency in both ears or 40-dB loss at 1- and 2-kHz in one ear; DSI: both measured or self-reported VI and HI).	/	Age, sex, race, education, past myocardial infarction, diabetes, hypertension, heart failure, and follow-up	9
Lam BL, 2006, America	116 796	7 (x̄)	Over 18	45.9	ICD-9.	ICD-9	Age, marital status, educational level, self-rated health, and number of nonocular and nonauditory conditions	6
Gopinath B, 2013, Australia	2812	10	Over 55	43.2	VI: 20/40 or worse in the better eye; HI: >25 dB at the PTA of 4 frequencies, 0.5, 1, 2, and 4 kHz, in the better ear; DSI: concurrent visual (either presenting or best-corrected) and HI.	ICD-9, ICD-10	Age, sex, body mass index, systolic blood pressure, current smoking status, poor self-rated health, walking disability, presence of hypertension and/or diabetes, history of cancer, angina, stroke and/or acute myocardial infarction, and cognitive impairment	9
Fisher D, 2014, America	4926	5	76.4 ± 5.5 (66–96)	43.1	HI: hearing level ≥35 dB for the PTA of 4 frequencies, 0.5, 1, 2, and 4 kHz, in the better ear with hearing aid removed; VI: ≤20/50 in the better eye assessed via an autorefractor.	ICD-10	Sex, age, smoking status, body mass index, hypertension, diabetes, self-reported health status, cognitive status, self-reported history of falls, total cholesterol, baseline CVD history, and hearing aid use	8
Liljas AE, 2016, Britain	3981	10	63–85	100	Self-reported	General practice records and mortality registers	Social class, obesity, smoking, physical activity, hypertension, and diabetes mellitus	6
Liu PL, 2016, America	3871	6	73.3	35.1	Self-reported	National death index	Age, sex, race, education, marital status, body mass index, history of smoking, depression, and a health index score that reflects self-reported disease burden	7
Miyawaki A, 2020, Japan	9522	9 (x̄)	40–69	/	Self-reported	Japan’s compulsory registration system	Age, sex, education years, living area, income level, marital status, primary occupation, self-rated health (self-reported histories of cancer, stroke, heart disease, diabetes, dyslipidaemia and hypertension), body mass index, smoking status, exercise habits, alcohol consumption, and dietary patterns	8
Zhang Y, 2020, China	8788	16	92.3 ± 7.6	39.9	Assessed by the interviewers	Interviews by contact family members	Age, sex, education years, ethnicity, marital status, co-residence, residence, economic independence, smoking status, drinking status, regular exercise, daily living disability, cognitive impairment, body mass index, and number of diseases	7
Sun J, 2020, China	37 076	21	/	41.4	Assessed by the interviewers	Acquired from family member or the village doctor	Age, sex, enrolment year, province, residence, ethnicity, marriage status, occupation, access to medical service, smoking status, drinking status, exercise status, activities of daily living score, physical performance score, Mini-Mental State Examination score, food diversity score, social activity score, chronic disease score	6
Zhang X, 2020, Britain	113 563	14	56.8 ± 8.09	45.5	VI: visual acuity worse than LogMAR 0.3; HI: speech-reception-threshold of over −5.5 dB in the participant’s better ear; DSI: one presented concurrent VI and HI.	National Health Service Central Register	Age, gender, ethnicity, education, Townsend index, obesity, smoking status, physical activity, history of hyperlipidaemia, hypertension, diabetes, cancer, and overall health status	8
Vohra V, 2024, America	1825	16	77.4 ± 3.2	48	VI: visual acuity, contrast sensitivity, and stereo acuity were assessed on the basis of the Bailey-Lovie, Pelli-Robson, and Frisby tests, respectively; HI: >25 dB at the PTA of 4 frequencies, 0.5, 1, 2, and 4 kHz, in the better ear.	/	Age, sex, race, education, smoking status, body mass index, clinic site, relevant comorbidities, and physical function as a time-dependent covariate	8

CVD – cardiovascular disease, DSI – dual sensory impairment, HI – hearing impairment, ICD – International Classification of Diseases, MD – median, NOS – Newcastle-Ottawa Quality Assessment Scale, PTA – Pure-Tone Audiometry, VI – vision impairment, x̄ – mean

### Risk of bias assessment

The quality of the included cohort studies was assessed by the NOS, and the results are provided in **Table 1**. Each one of the included studies were rated as moderate to high quality, with an average score of 7.2, indicating high quality in general.

### Risk of all-cause mortality

**Figure 2:**
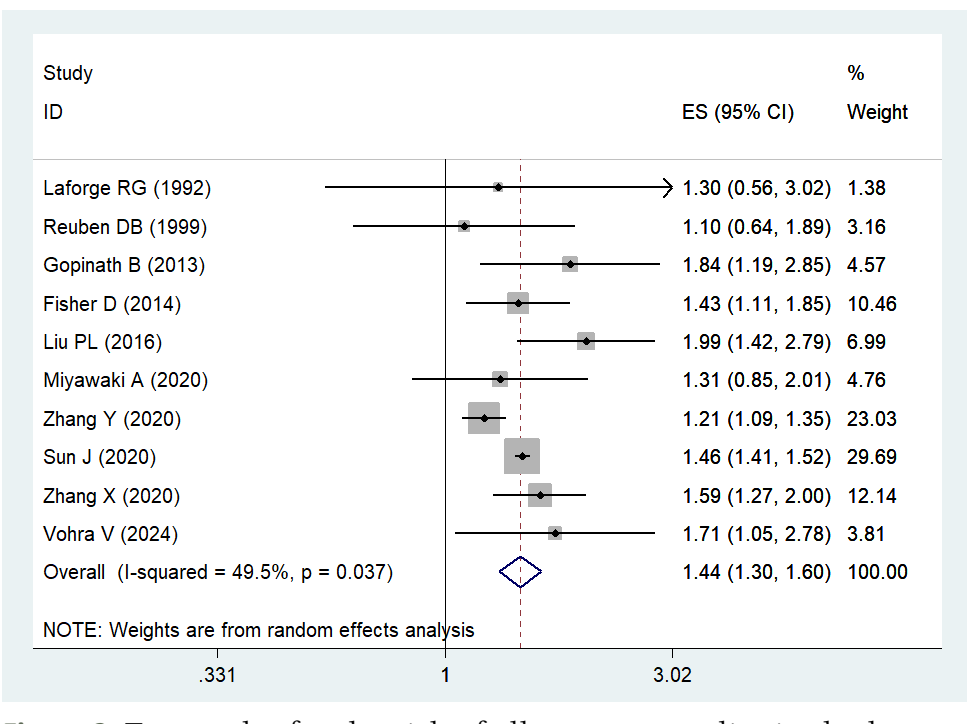
Forest plot for the risk of all-cause mortality in dual sensory impairment. CI – confidence interval.

**Figure 3:**
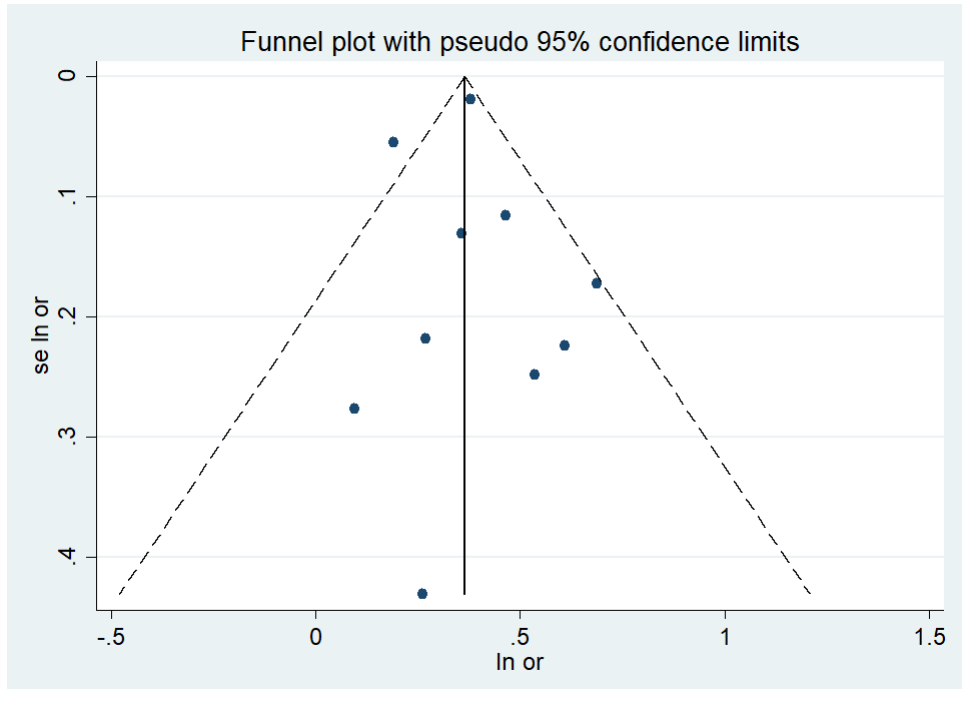
Funnel plot for all-cause mortality in dual sensory impairment patients.

Eleven studies reported the risk of all-cause mortality from DSI [26,27,29–37]. Pooling analysis indicated that DSI was associated with an increased risk of all-cause mortality (HR = 1.442; 95% CI = 1.303–1.596, *I*^2^ = 49.5%, *P* < 0.001) (**Figure 2**). Considering to the significant heterogeneity, we additionally carried out a sensitivity analysis to figure out the source of the heterogeneity. The sensitivity analysis demonstrated that none of the included studies modified the pooled effect size, indicating the robustness of the overall findings, as shown in Figure S1 in the **Online Supplementary Document**. A visual evaluation of the funnel plot showed no substantial publication bias (**Figure 3**). Egger's regression tests (*P* = 0.917) indicated that publication bias was insignificant. Patients with VI (HR = 1.166; 95% CI = 1.089–1.248, *I*^2^ = 45.6%, *P* < 0.001) or HI (HR = 1.207; 95% CI = 1.131–1.288, *I*^2^ = 51.86%, *P* < 0.001) only were also associated with an increased risk of all-cause mortality (Figure S2 in the **Online Supplementary Document**).

### Risk of cardiovascular mortality

**Figure 4:**
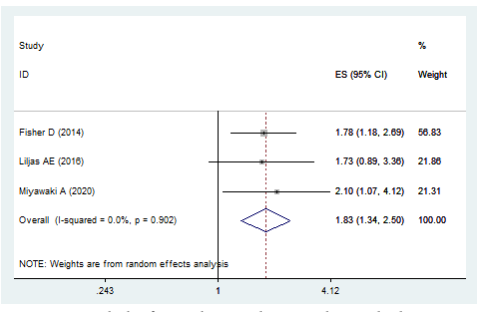
Funnel plot for cardiovascular mortality in dual sensory impairment patients. CI – confidence interval.

Three studies assessed the risk of cardiovascular mortality from DSI [30,31,33], and revealed that DSI had an increased risk of cardiovascular mortality (HR = 1.832; 95% CI = 1.343–2.500, *I^2^* = 0%, *P* < 0.001) (**Figure 4**).

### Subgroup analysis

Subgroup analyses were carried out on sex and territory type for all-cause mortality (**Table 2**). Subgroup analysis in sex type indicated that DSI were related to an increased risk of all-cause mortality in both male (HR = 1.468; 95% CI = 1.314–1.640, *I^2^* = 16.4%, *P* < 0.001) and female patients (HR = 1.352; 95% CI = 1.017–1.798, *I^2^* = 54.6%, *P* = 0.038). Whether in America (HR = 1.548; 95% CI = 1.285–1.866, *I^2^* = 8%, *P* < 0.001), Asia (HR = 1.336; 95% CI = 1.137–1.571, *I^2^* = 81.3%, *P* < 0.001), Europe (HR = 1.590; 95% CI = 1.267–1.995, *I^2^* = 0%, *P* < 0.001), or Oceania (HR = 1.840; 95% CI = 1.187–2.852, *I^2^* = 0%, *P* = 0.006), DSI all increased the risk of all-cause mortality.

**Table 2:** Results of subgroup analysis

Measures	All-cause mortality	
	**Pooled HR (95% CI)**	***P*-value**
Sex type		
*Male*	1468 (1314–1640)	<0.001
*Female*	1352 (1017–1798)	0.038
Territory type		
*America*	1548 (1285–1866)	<0.001
*Asia*	1336 (1137–1571)	<0.001
*Europe*	1590 (1267–1995)	<0.001
*Oceania*	1840 (1187–2852)	0.006

CI – confidence interval, HR – hazard ratio

## DISCUSSION

The meta-analysis included 12 cohort studies with 310 211 participants, showing that DSI patients faced a 1.442- and 1.832-fold higher risk of all-cause and cardiovascular mortality, respectively. Thus, recognising DSI as an independent risk factor for both all-cause and cardiovascular mortality is warranted.

Previous studies found a substantial relation between single sensory impairment, VI or HI, and mortality [38,39]. However, a limited number of studies concentrated on the relation between DSI and mortality, generating conflicting results [18,40]. Therefore, to achieve reliable estimates and address this divergence, in the present study, we set strict inclusion criteria that only the study with definite cohort design could be included. To the best of our knowledge, this is the first systematic review and meta-analysis, and the most comprehensive evidence-based synthesis to date, on the associations of DSI with all-cause and cardiovascular mortality. In agreement to prior reviews, our analysis found that DSI was associated with an increased risk of all-cause and cardiovascular mortality [19,41,42]. When HI and VI occur together, their combined relation with all-cause mortality is 1442-fold, which is higher than either HI or VI alone (1.166- and 1.207-fold, respectively). This result indicated that the combination of HI and VI could be related to an excess risk of mortality.

So far, the exact mechanisms about the relation between DSI and mortality remains uncertain [34]. But the underlying mechanisms could be summarised as following two aspects. First, DSI leads to a decreased functional ability and an increased vulnerability to external stressors since sympathetic activation [37,40]. Sensory impairment directly interferes with daily life activity, presenting a reduced mobility to receive health care services, and an increased risk of emotional damage and social isolation [43]. Notably, persons with DSI are remarkably more limited than those with just one sensory impairment as they cannot depend on their unimpaired sensory function to compensate [44]. Therefore, DSI patients are more likely to suffer from life-threatening accidents, such as fall and frailty [45]. Second, sensory impairment may be an indicator of multiple systemic processes, such as ageing of the overall cellular functioning, neural degeneration, immune-related senescence, or chronic inflammation [46–48]. And these pathological processes would cause hypercortisolism, endothelial dysfunction, hypercoagulability, or accelerated atherosclerosis, which are also strong predictors of all-case or cardiovascular mortality [40]. Therefore, compared with health individuals or single sensory impairment patients, DSI patients could signify a more progressed state of aging and a more serious degenerative condition [36].

We identified heterogeneity among the 12 studies, attributable to several factors. First, three studies had relatively small populations, each with fewer than 3000 participants, potentially impacting result accuracy [26,27,37]. Further research with larger sample sizes is necessary to establish associations. Second, disparities existed in diagnostic criteria for DSI and mortality, including variations in ICD-9/10 coding, interviewer assessment, and self-reporting. Additionally, most studies relied on electronic health records for diagnosis, which could have affected outcomes. Lastly, studies were conducted across diverse regions (America, Europe, Asia, and Oceania), with subgroup analysis revealing variations in mortality risk. Regional bias may stem from differences in economic development and education levels. However, due to limited original research, our analysis lacks the capacity to assess the DSI-mortality link within specific racial groups. Therefore, further studies involving homogeneous racial cohorts are necessary for clarification.

The present study consolidates findings on the link between DSI and mortality risk, suggesting DSI as an independent mortality risk factor. Emphasising the need to monitor all-cause and cardiovascular mortality risk in DSI patients. Despite many unanswered questions regarding the association of sensory impairments with mortality, these findings represent an opportunity for additional focus on the DSI patients. In terms of identification, we should explore a clinically accessible approach to identify vulnerable patients, and provide objective psycho-physical assessments for the at-risk populations. In terms of prevention, when DSI are encountered, interventions targeting mitigation strategies should be explored. Future studies could examine the effect of sensory training or impairment correction on mortality decline. For example, hearing aids or cochlear implantation, visual correction or surgery can be administrated to correct HI and VI, respectively.

However, the study has limitations. First, it only analyses 12 cohort studies. Although there was a risk of missing other types of studies, the methodological heterogeneity was well reduced. Second, specific mortality data (e.g. injury, infection, cancer) were lacking, precluding subgroup analysis by mortality type. Third, covariate analysis was not conducted, but since included studies adjusted for confounders, confounding bias was controlled. Nevertheless, further well-designed studies with larger sample sizes comparing cases with similar baselines are warranted.

## CONCLUSIONS

DSI patients are at higher risk of all-cause and cardiovascular mortality. This study underscores the importance of early, scientific intervention for DSI patients to mitigate mortality risk.

## Additional material


Online Supplementary Document
