# Supplementary material for: All-cause and cardiovascular mortality in dual sensory impairment patients: A meta-analysis of cohort studies
Source: J Glob Health. 2024 Nov 29;14:04258. doi: 10.7189/jogh.14.04258 (PMC11606439; doi:10.7189/jogh.14.04258)
Supplement: Online Supplementary Document [file jogh-14-04258-s001.pdf]

Appendix I

Details of the Literature Search Strategy

(1) PubMed (March 22, 2024)

Search	Query	Items found
#1	"Mortality"[MeSH Terms]	426,121
#2	"mortality*"[Title/Abstract] OR "death rate*"[Title/Abstract] OR "case fatality rate*"[Title/Abstract] OR "crude death rate*"[Title/Abstract] OR "crude mortality rate*"[Title/Abstract] OR "mortality decline*"[Title/Abstract] OR "mortality determinant*"[Title/Abstract] OR "differential mortality*"[Title/Abstract] OR "age specific death rate*"[Title/Abstract] OR "excess mortality*"[Title/Abstract]	1,053,061
#3	#1 OR #2	1,309,247
#4	"sensory impairment*"[Title/Abstract] OR "sensory*"[Title/Abstract]	223,940
#5	"Risk"[MeSH Terms]	1,405,029
#6	"risk*"[Title/Abstract]	3,062,494
#7	#5 OR #6	3,558,454
#8	#3 AND #4 AND #7	531

(2) Embase (March 22, 2024)

Search	Query	Items found
#1	'mortality'/exp	1,450,433
#2	mortality*:ab,ti OR 'death rate*':ab,ti OR 'case fatality rate*':ab,ti OR 'crude death rate*':ab,ti OR 'crude mortality rate':ab,ti OR 'mortality decline*':ab,ti OR 'mortality determinant*':ab,ti OR 'differential mortality*':ab,ti OR 'age specific death rate*':ab,ti OR 'excess mortality*':ab,ti	1,542,820
#3	#1 OR #2	2,016,058
#4	'sensory impairment*':ab,ti OR 'sensory*':ab,ti	274,044
#5	'risk'/exp	3,201,644
#6	risk*:ab,ti	4,383,485
#7	#5 OR #6	5,252,866
#8	#3 AND #4 AND #7	875

(3) Cochrane Library (March 22, 2024)

Search	Query	Items found
#1	MeSH descriptor: [Mortality] explode all trees	18,860
#2	(Mortality*):ti,ab,kw OR ("Death Rate*"):ti,ab,kw OR ("Case Fatality Rate*"):ti,ab,kw OR ("Crude Death Rate*"):ti,ab,kw OR ("Crude Mortality Rate*"):ti,ab,kw	120,839

#3	("Mortality Decline*"):ti,ab,kw OR ("Mortality Determinant*"):ti,ab,kw OR ("Differential Mortality*"):ti,ab,kw OR ("Age Specific Death Rate*"):ti,ab,kw OR ("Excess Mortality*"):ti,ab,kw	389
#4	#1 OR #2 OR #3	124,693
#5	MeSH descriptor: [Risk] explode all trees	56,283
#6	(risk*):ti,ab,kw	311,823
#7	#5 OR #6	315,725
#8	("Sensory Impairment*"):ti,ab,kw OR (Sensory*):ti,ab,kw	22,911
#9	#4 AND #7 AND #8	176

(4) Web of Science core collection (March 22, 2024)

Search	Query	Items found
#1	TS=("Sensory Impairment*") OR TS=("Sensory*")	1,130,061
#2	TS=(Mortality*) OR TS=("Death Rate*") OR TS=("Case Fatality Rate*") OR TS=("Crude Death Rate*") OR TS=("Crude Mortality Rate*") OR TS=("Mortality Decline*") OR TS=("Mortality Determinant*") OR TS=("Differential Mortality*") OR TS=("Age Specific Death Rate*") OR TS=("Excess Mortality*")	2,174,611
#3	TS=(risk*)	5,062,133
#4	#1 AND #2 AND #3	3,948
